# Natural Source of Drugs Targeting Central Nervous System Tumors—Focus on NAD(P)H Oxidoreductase 1 (NQO1) Activity

**DOI:** 10.3390/brainsci15020132

**Published:** 2025-01-29

**Authors:** Nikola M. Stojanovic, Milica Mitić, Jovan Ilić, Milica Radić, Miša Radisavljević, Marko Baralić, Miljan Krstić

**Affiliations:** 1 Department of Physiology, Faculty of Medicine, University of Niš, 18000 Niš, Serbia; 2 Center for Pathology, University Clinical Centre Niš, 18000 Niš, Serbia; stankovic.milica93@gmail.com (M.M.); krstic.miljan@gmail.com (M.K.); 3 Faculty of Medicine, University of Niš, 18000 Niš, Serbia; jovanilicneuro@gmail.com (J.I.); milica91nis@ymail.com (M.R.); radisavljevicmisa@gmail.com (M.R.); 4 Department for Neurosurgery, University Clinical Centre Niš, 18000 Niš, Serbia; 5 Department for Radiation Oncology, University Clinical Centre Niš, 18000 Niš, Serbia; 6 Faculty of Medicine, University of Belgrade, 11080 Belgrade, Serbia; baralicmarko@yahoo.com; 7 Department of Nephrology, University Clinical Centre of Serbia, 11000 Belgrade, Serbia; 8 Department of Pathology, Faculty of Medicine, University of Niš, 18000 Niš, Serbia

**Keywords:** brain tumors, NAD(P)H oxidoreductase, NQO1 protein, glioma, glioblastoma

## Abstract

Central nervous system (CNS) tumors involve a large and diverse group of malignancies that arise from various cell types within the brain tissue. Although there are advances in treatments, CNS tumors still remain challenging, due to their complex biology and the delicate nature of the surrounding tissue. NAD(P)H O=oxidoreductase 1 (NQO1) is an enzyme that plays a critical role in the detoxification of quinones, protecting cells from oxidative stress. In CNS tumors this enzyme is often overexpressed, which contributes to the resistance of tumor cells to chemotherapy by enhancing their antioxidant defenses. NQO1 influences the progression of CNS tumors by affecting downstream signaling pathways, such as those involving the transcription factor SNAIL, as well as others that are associated with tumor behavior. Plants represent a valuable source of numerous constituents with different chemical structures known to affect different molecular signaling pathways associated with different pathologies.

## 1. Introduction

Brain tumors are considered one of the deadliest forms of cancer, while glioblastoma stands out as the most aggressive type, with a median overall survival rate of 13.5 months following diagnosis [1]. In the pediatric population, brain tumors are the most common and lethal of all solid tumors, representing approximately 25% of all pediatric cancers. Survivors face the long-term consequences of treatment, including surgery and oncological treatment, which can damage the developing brain. These tumors are extremely difficult to treat due to their biological characteristics, which often make them inaccessible to surgery [2]. In addition, the presence of the blood–brain barrier (BBB) makes it difficult to deliver drugs to tumors and reduces the effectiveness of chemotherapy. Also, the unique properties of the brain, including genetic and epigenetic factors, often contribute to the resistance of these tumors to standard and new treatments [2,3].

## 2. Epidemiology

Brain tumors are the eighth most common cancer in adults over the age of 40. The most common benign tumors in adults older than 20 years are meningiomas and pituitary tumors, while malignant tumors, such as gliomas, are less common [4]. About 5–10% of brain and central nervous system (CNS) tumors are associated with a family history of the disease, and certain syndromes can increase the risk of these tumors [5]. Given that there are no clear environmental risk factors, research is focused on genetic predispositions, especially in patients with gliomas, which cause the most deaths from malignant brain tumors [4]. Glioblastoma, a very aggressive primary malignant tumor with a low overall survival, accounts for 14.5% of all CNS tumors and 48.6% of malignant CNS tumors [6]. They originate from astrocytic glial cells and represent a grade IV tumor, while their frequency varies depending on the source, ranging from 3.19 to 4.17 cases per 100,000 persons per year. However, they account for 3–15% of primary brain tumors in the pediatric population, with a frequency from 0.85 per 100,000 [6,7].

## 3. General Taxonomy

There have been changes in the diagnosis of CNS tumors, as advances in molecular genetics have revealed various features of these tumors. Some molecular changes were introduced into the diagnostic process of certain tumors, including histopathological and molecular data [8]. The 2021 World Health Organization (WHO) Classification of Tumors of the CNS largely follows the 2016 classification, with a chapter on gliomas and neuronal-glial tumors undergoing significant changes. In addition, tumor types common to other organ systems are grouped together, such as mesenchymal (non-meningothelial) tumors, melanocytic tumors, and those that are similar. A chapter on genetic tumors and syndromes has been added [8,9,10]. Thus, the WHO CNS 2021 classification includes molecular genetics with clinical relevance to a greater extent, and the latest edition includes elements of both histopathology and molecular genetics, thus creating a somewhat mixed taxonomy. This fifth edition builds on the previous edition and uses the recommendations to advance molecular diagnostics, but the combination of histology and molecular information remains at the heart of the CNS tumor classification [8,9] (Table 1).

## 4. Gliomas

Gliomas represent approximately 80% of primary malignant tumors in the CNS. The WHO employs a grading system for gliomas from grades 1 to 4, with grades based on histological features, such as cellular atypia, proliferation patterns, and the presence of necrosis. Diffuse astrocytomas, pilomyxoid astrocytomas, and pleomorphic xanthoastrocytomas (grade 2), as well as pilocytic astrocytomas and subependymal giant cell astrocytoma (grade 1), are considered low-grade gliomas, while oligodendrogliomas and oligoastrocytomas belong to the low-grade oligodendroglial tumors (grade 2). The revised classification of diffuse gliomas now considers the IDH 1/2 mutation status and 1p/19q codeletions, which is expected to abolish the oligoastrocytoma category and redefine gliomatosis cerebri as a growth pattern [10]. Diffuse gliomas are the most common tumors of the central nervous system in adults, and patient survival depends on the specific subtype and histological grade of the tumor [11]. The development of an integrated classification that takes into account morphological and molecular features, such as IDH gene mutations and chromosome 1p/19q codeletions, allows for a more accurate diagnosis and a better assessment of the prognosis. According to the 2021 WHO classification, diffuse gliomas in adults are now divided into three main types: astrocytoma with IDH mutations, oligodendroglioma with IDH mutations and 1p/19q codeletions, and glioblastoma without IDH mutations (Table 2) [12].

Currently, the recommended treatment for gliomas involves maximum safe tumor resection, radiotherapy, and the administration of temozolomide [13]. However, due to the invasive nature of glioma, tumor recurrence is almost inevitable as it is impossible to completely remove all tumor cells. Accuracy in determining tumor boundaries during surgery can improve treatment outcomes and preserve quality of life, but resection is difficult due to the complex composition of tumor boundaries that include tumor cells, reactive glial, and immune cells, as well as healthy brain cells [14,15]. Modern intraoperative optical techniques, such as fluorescence-guided surgery, enable more precise labeling of tumor cells and a clearer definition of the border between the tumor and healthy tissue, which can contribute to safer tumor removal [13]. Awake surgery is the gold standard for brain mapping, as it is the only technique that allows for the direct location of neural networks. Recent studies show that the combination of awake mapping with real-time neuropsychological testing enables a higher resection rate and the preservation of quality of life in patients with gliomas affecting language and eloquent functional networks, while modern imaging techniques and advanced surgical tools further increase the possibilities for resections in the eloquent zones [16]. Awake resection management allows neurosurgeons to check the patient’s functional abilities during surgery and use electrical stimulation to temporarily disrupt or activate key areas, thereby reducing the risk of damage and increasing the chance of a better outcome [13,16]. Advanced imaging techniques, such as intraoperative MR, ultrasonography, and neurostimulation during awake craniotomy, have become essential to increase the precision of glioma resection while preserving neurological functions [17,18].

## 5. Brain Metastases

Brain metastases (BMs) are often a sign of poor prognosis in patients with systemic cancer, and overall survival is usually circa 6 months [19]. The exact frequency of BMs may vary, and most often, they are caused by lung cancers (30–60%), breast cancers (15–20%), and skin (5–10%) and gastrointestinal tract cancers (4–6%) [20,21]. The treatment of BMs aims to control the disease in the CNS, prevent neurological complications, and prolong life, but the optimal treatment is determined individually, taking into account various factors, such as the tumor histology, the number of lesions, and the general condition of the patient, such as the Karnofsky Performance Scale and Neurological Assessment of Neuro-Oncology Scale [19,20,22]. Techniques that help the neurosurgeon in precise tumor removal, preserving the function of eloquent zones and reducing postoperative complications, are neuronavigation, the applications of diffusion tensor imaging and tractography, subcortical stimulation, and fluorescence-guided resection. The application of these methods improves local disease control, but individualization of the approach is still critical [20,23,24].

## 6. Brain Tumor Histopathology

Today, the pathohistological approach is based on the analysis of histological features, such as the cell morphology and mitotic activity, of complex tumors. Besides that, the immunological profiling of tumor cells helps in determining the cellular differentiation/tumor origin. The aggressive potential of brain tumors, especially glioblastomas (GBMs) often results from their intratumor heterogeneity owing to various differentiated tumor cell populations. This cell heterogeneity is also the result of interactions among tumor, endothelial, glial, and inflammatory cells and pericytes, which are an essential part of the tumor microenvironment. The malignant potential of CNS tumors is based on their histomorphological features/pleomorphism, mitotic activity, vascular proliferation, and necrosis [25]. Besides histopathological tumor profiling, the molecular approach is increasingly prevalent for the identification the molecular/genetic sites disrupted in brain tumors. Leading molecular markers, such as IDH1 and IDH2 mutations and codeletions in 1p and 19q chromosomes [26], divide gliomas into three molecular different subtypes: IDH-mutant (1p19q codeleted), IDH-mutant (1p19q intact) and IDH-wild-type gliomas [27]. Point mutations in IDH1/IDH2 are mostly present in diffuse astrocytoma WHO grade II and III and non-diffuse gliomas. On the other hand, glioblastomas with IDH1/IDH2 mutations are defined as “secondary” glioblastomas, which arise from low-grade diffuse gliomas, while IDH-wild-type is considered “primary” or “de novo” glioblastoma [27]. Furthermore, IDH-mutant glioblastomas show longer overall survival compared to IDH-wild-type tumors [27,28].

Gliomas, according to their histomorphological features, are divided into two major groups: diffuse and non-diffuse gliomas. Diffuse glioma cells tend to migrate through the brain parenchyma, which can make them inaccessible for surgical manipulation/treatment. Therefore, diffuse astrocytoma has the potential to progress into high-grade gliomas during 5–6 years [29]. According to the tumor cell origin, diffuse gliomas include astrocytoma with clumped chromatin and ungulated nuclei, oligodendrogliomas—with round, uniform nuclei, and oligoastrocytomas, originating both from astrocytes and oligodendroglia cells with an intermediate cellular morphology [30]. Compared to the diffuse group, non-diffuse gliomas are more clearly demarcated from the environment as in the case with pilocytic astrocytoma and different types of ependymoma grouped in this tumor category [25]. IDH-mutant astrocytomas are the most frequent in clinical practice. Histomorphologically, astrocytoma graded as CNS WHO grade 2 shows variable cellular atypia and irregular nuclei, somewhere, and glial processes, but without necrosis and microvascular proliferation. CNS WHO grade 3 astrocytomas are characterized by various cellular morphologies, as elongated or eccentric astrocytic nuclei, a glassy eosinophilic cytoplasm (gemistocytes), large pleomorphic cells or small cells with scant cytoplasm, and fibrillar glial processes. Mitotic figures are also present, while necrosis and vascular proliferation are absent. On the other hand, emphasized cellular atypia/pleomorphism (hypercellular pattern), an increased number of mitotic figures, necrosis, and vascular proliferation are major criteria (Saint Anne—Mayohistological criteria) for diagnosing astrocytoma CNS WHO grade 4 [27,30,31].

Brain tissue is consisting of a milieu of cells, such as neurons, astrocytes, glial cells, and oligodendrocytes, including populations present in all human tissue, such as macrophages (microglia in CNS) and endothelial and inflammatory cells. The behavior of these cell populations, including blood–brain barrier (BBB) permeability, represents part of the tumor microenvironment which is very important for tumor progression, proliferation, and metastasis. Furthermore, tumor-infiltrating neutrophils can stimulate tumor-initiating cell (TIC) proliferation through the production of S100-protein, which is important for the pathogenesis of glioblastoma. According to that, IDH-mutant gliomas have a lower level of neutrophils compared to low-grade gliomas. Interestingly, in mesenchymal tumors, an increased number of macrophages was found to be associated with worse prognosis. Low-grade gliomas have a lower density of inflammatory cells and reduced secretion of cytokines. Also, the extracellular matrix in brain tumors shows high cellular density, which causes/contributes to tissue hypoxia and aggressive tumor behavior [28]. Most brain tumor cells create strong malignant synaptic contacts (malignant synaptogenesis) through which they send signals important for proliferation and further brain/tumor invasion [32].

The tumor microenvironment is conditional on hypoxia, neoangiogenesis, the BBB, and reactive oxygen species (ROS). Appropriate levels of ROS can initiate and stimulate the progression of brain tumors, partly affecting the immune cells from the environment through the activation of growth signaling pathways. On the other hand, the accumulation of ROS products often causes the death of glioma cells, as does the depletion of the “ROS function“ [33]. Glioma stem cells (GSCs), also known as tumor-initiating cells, represent very resistant immune system cells, which have a chance of further tumor development and the potential for invasiveness and recurrence. GSCs interact with non-tumor cells from the tumor microenvironment using the immune cytokine receptors, such as interleukin 6 (IL-6), vascular endothelial (VEGF), and epidermal growth factor (ERGF), notch signaling pathways, and platelet derived growth factor, through which tumor cells avoid the immune response [34]. According to that, tumor cells create a perivascular niche, with endothelial cells providing the conditions for neoangiogenesis and, therefore, tumor growth/development. ROS-sensitive signaling pathways have a critical role in cellular metabolism, inflammatory processes, and angiogenesis, which suggests their role in tumor proliferation and growth [34,35].

## 7. NAD(P)H NQO1 in Brain Tumors

NAD(P)H:quinone oxidoreductase 1 (NQO1) is an antioxidant flavoenzyme important for cell protection from a wide range of substrates, resulting from oxidative stresses [36]. This enzyme represents a homodimer with one FAD linked per monomer [37]. NQO1 inhibits redox cycling by minimizing the reaction between quinones and semiquinone substrates, which disrupts the production of superoxide radicals and prevents carcinogenesis [38,39,40]. NQO1 is predominantly located in the cytosol (about 90%) but also exists in the nucleus, mitochondria, and endoplasmic reticulum [38,41,42]. This cytoprotective enzyme is mostly present in various epithelial cells, the endothelium, and adipocytes. Interestingly, human liver tissue (hepatocytes) does not express NQO1, unlike the preneoplastic lesions seen in liver cancers and others, such as breast, pancreas, lung, and colon cancer [37,38,40]. On the other hand, the NQO1 concentrations present in normal, healthy cells often are not enough to initiate apoptosis [41]. The overexpression of NQO1 provides cell protection by decreasing the levels of reactive quinones and quinone-imines and transforming the less detrimental and less reactive hydroquinone substrates [38]. This cytoprotective enzyme is responsible for catalyzing the reduction of two electrons from quinones to hydroquinones [36,39,43]. Furthermore, low cell expression of NQO1 can potentially provoke the occurrence of cancer, especially when it is associated with smoking or exposure to benzene. Also, NQO1 interacts with and stabilizes tumor suppressor genes, p53 and p73, which means that NQO1 protects cells from carcinogenesis through the regulation of proteasomal degradation [40,44,45].

In addition to different pH values, the tumor microenvironment manifests different oxido-reductive potentials, which can cause the malignant transformation of/alterations to normal cells through the variable expression of enzyme activity. According to that, NQO1 may be crucial for the in situ activation of prodrugs that impact cancer cells with high levels of NQO1. Also, NQO1 can initiate mitochondria- and ER-mediated apoptosis through BAS-activated regulation [41].

In healthy/human brain tissue, NQO1 is mainly expressed in astrocytes, some oligodendrocytes, and rarely narrow neuronal subpopulations [39]. In rodents, the expression of NQO1 has been found in the mesencephalon in cells like substantia nigra neurons, dopaminergic neurons, and also in human substantia nigra neurons frequently exposed to oxidative stress [43,44]. Resistant GBM often has acquired mutations in NQO1without adequate bound FAD cofactors, which makes NQO1 catalytically inactive [37]. The implication of the transcription factor Nrf2 in neuronal NQO1 expression explains that low basal levels of Nfr2 are co-related to deficits of neuronal NQO1 in appropriate brain cells [39]. The activation of NQO1 can nullify the harmful effects of ROS on GBM cells, and thus, the overexpression of NQO1 significantly increases tumor growth. On the other hand, the depletion of NQO1 significantly decreases cellular proliferation through an increase in the ROS levels [46,47]. Some authors suppose that the NQO1-mediated necrosis triggered by quinones can induce NQO1-dependent programmed cell death caused by oxidative stress, which suggests that this mechanism can be implement as a potential therapeutic treatment [48]. The term for cell death mediated by the NQO1 system is suggested to be noptosis [47]. Keeping in mind the presence of NQO1 in cancer cells, potential therapeutic treatments, such as the inhibition of NQO1, could/should be considered [44].

## 8. Plant-Based Therapy Targeting CNS Tumors

Bearing in mind the role of NQO1, there are two strategies that could be pursued with this enzyme being a central target of chemotherapeutics. One strategy is aiming to increase the expression of NQO1, while the other one is to inhibit this enzyme. Its inhibition could affect cell viability and functions, and this is especially true for the GBM cells expressing this enzyme in large amounts [46,47]. In a recent review paper, ROS-mediated strategies for treating GBM with plants and the compounds originating from them were discussed; however, there was no mention of the NQO1 system as a potential target [49]. Although ROS is one of the initiators of cell damage, further cascades of events lead to immunogenic cell death through the activation of dendritic cells and CD8+ T cells [50,51].

Medicinal plants have been utilized for centuries in traditional medicine systems worldwide for their therapeutic properties. The systematic collection of data on medicinal plants not only helps to preserve traditional knowledge but also supports the development of new drugs and sustainable healthcare practices. In a recent review, the role of essential oil constituents in microglia functioning has been discussed, pointing to medicinal plant utility in the treatment of certain disorders [52]. Understanding their applicability and true potential involves collecting and documenting knowledge from various sources, both in the field (data from indigenous practices, ethnobotanical studies) and in laboratory settings, through precisely guided scientific research. Also, through this process, the identification of crucial active compounds is achieved, and further, their safety, effectiveness, and mechanisms of action could be validated. The search for potential plant-originating molecules was performed using SCOPUS and ScienceDirect base with key words that included “NQO1”, “glioma”, “glioblastoma”, and “plant” “downregulated”, yielding roughly 200 publications. Each publication was analyzed, and the results describing the inhibitory potential of tested plant constituents are presented.

Heme oxygenase-1 (HO-1), normal human bronchial epithelial cells (NHBEs), immortalized human bronchial epithelial cells (HBECs), lung adenocarcinoma cells (A549), pancreatic cancer (PANC-1 cells), silent mating-type information regulation 2 homolog 3 (Sirt3), Toll-like receptor 4 (TLR4), multidrug resistance protein 1 (MRP1).

Out of the total number of analyzed publications, 18 were found to deal with the plant constituents causing the downregulation in NQO (Table 3). In total, 17 plant-derived chemicals belonging to different classes produced the desirable activity in different, mainly in vitro cell, models. This activity was dominantly shown in non-CNS cancer cells, such as prostate, breast, and melanoma (Table 3), which aberrantly express NQO. Thus, additional experiments confirming their impact on CNS cancer cells is needed, even though the present findings give a promising starting point for such studies.

Some plants with a rich history of traditional usage in Chinese medicine were proven to be rich in constituents affecting the CNS response to damage, and the experiments conducted using the extracts of pure compounds further strengthened the connection between the origin of active molecules and tumors as targets. One such constituent, trilobatin, proved to be effective in silencing the Nrf2 and NQO1 pathways both in vitro, in a model of isolated rat astrocytes or cortical neurons, and in an in vivo model of focal cerebral ischemia [63]. The effects were found to be associated, in part, with ROS and the different systems associated with it [63]. These findings suggest that there are some relatively rare plant constituents that might carry significant potential for targeting NQO1. Furthermore, these results suggest that there is a need for further constant research in this field, aiming to find such molecules.

One of the compounds for which activity has been shown in various cancer cell models is plumbagin, a naphthoquinone found dominantly in plants belonging to Plumbago species (*P. indica* and *P. zeylanica*) and in *Diospyros kaki* but also in those of the Ancestrocladaceae, Dioncophyllaceae, Droseraceae, and Ebenceae families [66]. Plumbagin’s unique anticancer mechanisms include the modulation of signaling pathways, such as NF-κB, STAT3, MMP-9, VEGF, and Akt, as well as the induction of apoptosis, autophagy, and regulation of the cell cycle. Additionally, it promotes ROS generation, leading to oxidative DNA damage. Notably, it has the ability to sensitize cancer cells that are resistant to chemotherapy and radiotherapy [66]. For the scope of this review, the focus of plumbagin’s activity is on its action in different GBM cells lines and in an in vivo model of implanted GBM in rats and mice [60]. The activity of plumbagin in glioma cell lines (U251, U87, C6, and GL261) is mediated by the alteration of various signaling pathways, with predominance of the ferroptosis pathway. Also, pathways associated with cystine-glutamate antiporter (xCT) and glutathione peroxidase 4 (GPx4) were affected in cells exposed to plumbagin [60]. The interaction of plumbagin with NQO1 is rather specific, proven both using docking and in vitro interactions, acting as a bioactivatable drug [60]. Despite its strong anticancer potential, plumbagin has not yet been applied clinically due to challenges like its high lipophilicity, poor water solubility, short half-life, and low melting point [66].

The interplay between NQO1 inhibitors and chemotherapeutic-drug-resistant cancers has been revealed as well. In the case of NQO1 inhibition in cisplatin-resistant head and neck tumor cells, their sensitivity towards artesunate increases [67]. The specific results of the inhibition of NQO1 in cancer cells has been shown in the case of berberine, where this compound promoted immunogenic cell death after causing the increased production of ROS and cell oxidative damage [51]. The activation of cell interplay after ROS production involves the elevation of the levels of damage-associated molecular patterns (DAMPs) and subsequently the activation of dendritic and CD8 + T cells in vitro and in vivo. These results pinpointed that berberine is molecule with potential for the development of tumor vaccines and to serve as a novel therapeutic approach in the future [51]. Similar potential to increase sensitivity towards doxorubicin in breast MCF-7 cancer cells was observed for chrysin and chrysin-loaded nanostructured lipid carriers [56]. This study, apart from addressing the problem of the NQO1 system as a cancer target, aimed to improve the availability of chrysin, which might be a challenge in designing and implementing new treatments.

Modern approaches in treating cancers involve applying combination of chemotherapeutics and radiotherapy in order to achieve adequate results in suppressing tumor growth, preventing metastases, etc. An important quinone compound, β-lapachone, proved to be useful in abolishing radiation-induced increases in NF-κB activation and thus decreasing genes, such as bcl2, gadd45β, and cyclinD1. This activity of β-lapachone was mediated by the interaction between NQO1, which is known to activate NF-κB; therefore, this quinone inhibits the transcription of the survival signals needed for irradiated cells to survive [53].

There are some compounds present in all plants, such as quercetin, which deserve a deeper analysis and interpretation in the light of the NQO1 system. Namely, quercetin, alone [59] or in combination with vitamin C [56], downregulated NQO1 in MCF-7 breast cancer and PC3 prostate cancer cells, respectively. These findings suggest the usefulness of widely present polyphenols in decreasing this enzyme activity and its usage in GBM. On the other hand, this enzyme was found to be upregulated in some other types of cancers, CWR22Rv1 cells, exposed to quercetin [68]. The potential of quercetin should be checked in a model of GBM with aberrant NQO1 expression in order to further corroborate the potential of this naturally occurring compound. The discrepancy of the impact on the NQO1 system has also been reported previously for various *Glycyrrhiza* species (*G. glabra* L., *G. uralensis* Fisch. ex DC., and *G. inflata* Batalin) extracts in hepatoma and breast cancer cells [69].

One of the main issues with drugs aiming to treat all CNS tumors is the BBB, which is known to pose a great issue in chemotherapeutic approaches [2,3]. This is a highly selective, semi-permeable structure composed of tightly joined endothelial cells lining the brain’s capillaries, maintaining CNS homeostasis by regulating the passage of ions, nutrients, and waste products, while restricting toxins and pathogens [70]. Interestingly, the potential of a drug to pass the BBB in cases of targeting tumors, e.g., GBM, might not be the issue, since the tumor process is known to affect the membrane function. In the case of GBM, the tumor tissue increases BBB fluidity by decreasing the expression of claudin-5 and occludin [71]. In this way, the drugs with lesser penetrant potential might go across the BBB and target the tumor tissue. On the other hand, the potential molecules presented here are highly lipophilic, e.g., plumbagin, which can easily cross the BBB.

Also, it should be highlighted that potentially by decreasing NQO1 expression, there is a risk of subsequent damage to healthy tissue due to more ROS generation. This point of view is applied in the design of many drugs that are able to induce NQO1 and thus decrease ROS and prevent carcinogenesis and aid the organism fight against cancer. Many of these molecules can be found in natural sources, e.g., broccoli [72]. Thus, when designing and potentially applying the drugs targeting NQO1, these facts should be taken into account.

## 9. Conclusions

The NAD(P)H oxidoreductase 1 (NQO1) enzyme plays a crucial role in protecting cells from oxidative damage but is also involved in the resistance mechanisms of CNS tumors. The overexpression of NQO1 in these tumors supports cancer cell survival by reducing oxidative stress, enabling drug resistance, and facilitating tumor progression. Due to these effects, NQO1 has emerged as a promising target for novel CNS tumor therapies. Naturally occurring compounds, including those from traditional plant medicines, show potential in modulating NQO1 activity. Some research studies have focused on identifying both inhibitors and activators of NQO1 from natural sources, with inhibitors showing particular promise in increasing cancer cell susceptibility to treatment. The therapeutic applications of such molecules could potentially complement existing treatments, targeting NQO1’s unique role in CNS tumor resistance to therapy and enhancing treatment efficacy. One of the molecules pin-pointed in the up-to-date conducted studies is plumbagin, which has been studied both in vitro and in vivo, proving its potential against CNS tumors. However, further studies, both preclinical and clinical, are needed in order to adequately investigate the potency of some molecules targeting the NQO1 system.

## Figures and Tables

**Table 1:** 2021 WHO Classification of Tumors of the Central Nervous System: groups of tumors [10].

Gliomas, Glioneuronal and Neuronal Tumors
Choroid plexus tumors
Embryonal tumors
Pineal tumors
Cranial and paraspinal nerve tumors
Meningiomas
Mesenchymal, non-meningothelial tumors involving the CNS
Melanocytic tumors
Haematolymphoid tumors involving the CNS
Germ cell tumors
Tumors of the sellar region
Metastases to the CNS
Genetic tumor syndromes involving the CNS

**Table 2:** 2021 WHO Ccassification of gliomas, glioneuronal, and neuronal tumors [10].

Tumor Group	Tumor Types
Adult-type diffuse gliomas	Astrocytoma, IDH-mutant
	Oligodendroglioma, IDH-mutant, and 1p/19q-codeleted
	Glioblastoma, IDH-wild-type
Pediatric-type diffuse low-grade gliomas	Diffuse astrocytoma, *MYB*- or *MYBL1*-altered
	Angiocentric glioma
	Polymorphous low-grade neuroepithelial tumor of the young
	Diffuse low-grade glioma, MAPK pathway-altered
Pediatric-type diffuse high-grade gliomas	Diffuse midline glioma, H3 K27-altered
	Diffuse hemispheric glioma, H3 G34-mutant
	Diffuse pediatric-type high-grade glioma, H3-wild-type and IDH-wild-type
	Infant-type hemispheric glioma
Circumscribed astrocytic gliomas	Pilocytic astrocytoma
	High-grade astrocytoma with piloid features
	Pleomorphic xanthoastrocytoma
	Subependymal giant cell astrocytoma
	Chordoid glioma
	Astroblastoma, *MN1*-altered
Glioneuronal and neuronal tumors	Ganglioglioma
	Desmoplastic infantile ganglioglioma/desmoplastic infantile astrocytoma
	Dysembryoplastic neuroepithelial tumor
	Diffuse glioneuronal tumor with oligodendroglioma-like features and nuclear clusters
	Papillary glioneuronal tumor
	Rosette-forming glioneuronal tumor
	Myxoid glioneuronal tumor
	Diffuse leptomeningeal glioneuronal tumor
	Gangliocytoma
	Multinodular and vacuolating neuronal tumor
	Dysplastic cerebellar gangliocytoma (Lhermitte-Duclos disease)
	Central neurocytoma
	Extraventricular neurocytoma
	Cerebellar liponeurocytoma

**Table 3:** Phytochemicals decreasing NQO1.

Compound (Reference)	Class	Model	Effects
Berberine [51]	Alkaloid	B16F10 and A375 melanoma cells	Inhibits NQO1 activity
Beta-lapachone [53]	Quinone	Radiosensitized lung cancer A549 cells	Inhibits NF-kB activation mediated by NQO1
Brusatol [54]	Quassinoid	Myeloid leukemia, MCF-7 and MDA-MB-231, PANC-1 cells	Inhibits the expression of Nrf2-downstream genes—HO-1, NQO1, VEGF, and AKR1C
Caryophyllene oxide [55]	Sesquiterpenoid epoxide	HCCLM3 and HUH7 liver cancer cells	Downregulation of NRF2, FTH1, HO-1, NQO1, and GPX4
Chrysin [56]	Flavonoid	Breast MCF-7 cancer cells	Decreased Nrf2, NQO1, multidrug resistance-associated protein 1 (MRP1), and HO-1 mRNA
Gallic acid [57]	Phenolic acid	Cal33 and FaDu—head and neck carcinoma cells	Induction of apoptosis through upregulation of Bax and caspase-3 and downregulation of Bcl-2, NRF2, NQO1, and GCLC
Luteolin [56]	Flavonoid	NSCLC A549 cells	Decreases heme oxygenase 1 (HO-1), aldo-keto reductase 1C (AKR1C), and glutathione S-transferase Mu 1 (GSTm1) and glutathione levels
Neferine [58]	Bisbenzylisoqui-noline alkaloids	Human thyroid cancer cell lines IHH-4(JCRB1079) and CAL-62 (CL-0618)	Decreased relative protein expression of SLC7A11, GPX4, Nrf2, HO-1,and NQO1
Quercetin [59]	Glycoside	MCF-7 breast cancer cells	Decreases NQO1 and MRP1
Quercetin and vitamin C [56]	Glycoside, hydro soluble vitamin	Prostate PC3 cancer cells	mRNA and proteins of Nrf2, HO-1, and NQO1
Plumbagin [60]	Naphthoquinone	In vitro—human glioma cell lines (U251 and U87), rat glioma cell line (C6), mouse glioma cell line (GL261) In vivo—rat and mouse implantation model	Downregulates protein and mRNA levels of xCT and GPX4, interacts with NQO1
Sanguinarine [61]	Benzophenanthridine alkaloid	Zebra fish embryotoxicity	Oxidative stress and apoptosis-related genes with a decrease in genes of nrf2 and NQO1
Tannic acid [62]	Polyphenol	Mouse liver and kidneys	Decrease enzyme activity
Trilobatin [63]	Aryl beta-D-glucoside	In vitro: isolated rat astrocytes and cortical neurons, in vivo: focal cerebral ischemia	Decreases expression of TLR4, Nrf2, NQO1, and Sirt3
Trigonelline [64]	Alkaloid	In vitro model of oxaliplatin-induced colon cancer cell apoptosis	Enhances suppression of Nrf2 and major downstream target genes HO-1, NQO1, and MRP1
3′,4′,5′,5,7-Pentamethoxyflavone [65]	Flavonoid	Lung cancer A549 cells	Decreased Nrf2 expression and the translation of HO1 and NQO1

## Data Availability

The original contributions presented in this study are included in the article. Further inquiries can be directed to the corresponding author.
